# Technical efficiency of primary health units in Kailahun and Kenema districts of Sierra Leone

**DOI:** 10.1186/1755-7682-4-15

**Published:** 2011-05-11

**Authors:** Joses M Kirigia, Luis G Sambo, Ade Renner, Wondi Alemu, Santigie Seasa, Yankuba Bah

**Affiliations:** 1 World Health Organization Regional Office for Africa, B.P. 06, Brazzaville, Congo; 2 World Health Organization Country Office, P.O. Box 529, Freetown, Sierra Leone; 3 Ministry of Health and Sanitation, Freetown, Sierra Leone

## Abstract

**Background:**

The objectives of the study reported in this paper were to (i) estimate the technical efficiency of samples of community health centres (CHCs), community health posts (CHPs) and maternal and child health posts (MCHPs) in Kailahun and Kenema districts of Sierra Leone, (ii) estimate the output increases needed to make inefficient MCHPs, CHCs and CHPs efficient, and (iii) explore strategies for increasing technical efficiency of these institutions.

**Methods:**

This study applies the data envelopment analysis (DEA) approach to analyse technical efficiency of random samples of 36 MCHPs, 22 CHCs and 21 CHPs using input and output data for 2008.

**Results:**

The findings indicate that 77.8% of the MCHPs, 59.1% of the CHCs and 66.7% of the CHPs were variable returns to scale technically inefficient. The average variable returns to scale technical efficiency was 68.2% (SD = 27.2) among the MCHPs, 69.2% (SD = 33.2) among the CHCs and 59% (SD = 34.7) among the CHPs.

**Conclusion:**

This study reveals significant technical inefficiencies in the use of health system resources among peripheral health units in Kailahun and Kenema districts of Sierra Leone. There is need to strengthen national and district health information systems to routinely track the quantities and prices of resources injected into the health care systems and health service outcomes (indicators of coverage, quality and health status) to facilitate regular efficiency analyses.

## Background

The vision of Sierra Leone is to have a functional national health system delivering efficient, high quality health services that are accessible, equitable and affordable for everyone [1]. The general objective of the National Health Sector Strategic Plan (NHSSP) is to strengthen the functions of the national health system so as to improve access to health services (i.e. their availability, utilization and timeliness), quality of health services (i.e. their safety, efficacy and integration), equity in health services (particularly their access by disadvantaged groups), efficiency of service delivery (i.e. value for resources) and inclusiveness (partnerships) [2] in their delivery.

The first principle stated in the NHSSP [[2]:p.11] calls for "Accountable central governance and provision of effective and efficient local health services composed of a comprehensive range of primary and secondary health services across the nation". One of strategic objectives of the country's health care delivery is to increase attendance at health care facilities by mothers and children, the poor and other vulnerable groups from the current low level of 0.5 health facility contacts per person per year to 3 contacts per person per year by 2015.

The government of Sierra Leone is implementing several health sector reforms to improve efficiency of health services. These reforms deal with decentralization and devolution of authority to 19 local councils that are now responsible for managing the delivery of both the primary and secondary health care levels, transfer to the local councils of tied grants amounting to a quarter of the national health budget [2], introduction of user fees in public health facilities [3], and experimentation with autonomy for hospitals [4].

One of the three objectives of the Sierra Leone health financing strategy is to ensure equitable and efficient allocation and use of health sector resources by (i) developing and implementing equitable, needs-based criteria for allocating financial resources, (ii) harnessing NGO and private sector resources through contractual arrangements in pursuit of national health development goals, (iii) developing provider (health facilities and health workforce) payment mechanisms that create incentives for greater effectiveness and efficiency, and (iv) institutionalizing health sector efficiency monitoring [2]. The broad aim of this study was to contribute towards objective (iv) of the health financing strategy.

The 2005 Sierra Leone efficiency study applied the data envelopment analysis (DEA) approach to assess the technical efficiency (TE) of peripheral health units (PHUs) in Pujehun district [5]. The study reported in this paper is the second attempt to apply the DEA approach in measuring the TE of health units in the country.

The specific objectives of the study reported in this paper were:

• To estimate the TE of samples of community health centres (CHCs), community health posts (CHPs) and maternal and child health posts (MCHPs) in two districts of Sierra Leone;

• To estimate the output increases needed to make inefficient CHCs, CHPs and MCHPs efficient;

• To explore the strategies that increase TE of primary health care units.

### Review of literature on efficiency of primary health care units

The studies reviewed below demonstrate that the DEA approach has been fruitfully used in Africa, Europe and North America to monitor and evaluate efficiency of various primary health care decision-making units (DMUs).

Sebastian and Lemma [6] estimated the TE of 60 health posts in rural Tigray, Ethiopia. The inputs were number of health extension workers (HEWs) and of voluntary health workers (traditional birth attendants and community health workers). The outputs for each health post were health education sessions given by HEWs, pregnant women who completed three antenatal care visits, child deliveries, number of persons who repeatedly visited the family planning service, diarrhoeal cases treated in children under-five, visits carried out by community health workers, total new patients attended, and malaria cases treated. The mean scores for technical and scale efficiency were 0.57 (SD = 0.32) and 0.95 (SD = 0.11), respectively. Fifteen (25%) health posts were found to be technically efficient and 38 (63.3%) were operating at their most productive scale size.

Halsteinli, Kittelsen and Magnussen [7] used DEA-Malmquist indices to assess productivity growth in an unbalanced panel of 48-60 Norwegian outpatient child and adolescent mental health service units (CAMHS) over the period 1998-2006. Input variables were full-time equivalent (FTE) university-educated personnel (psychiatrists and psychologists) and FTE college-educated personnel (mainly from the fields of social work and education and psychiatric nurses). The outputs were treated patients and direct and indirect consultations. This study estimated three models: *Model P*, an unadjusted model where output was measured as total number of patients; *Model PGR*, incorporating case-mix adjustment where patients were split into eight groups believed to be clinically meaningful; and *Model PDG_C*, in which the aggregate numbers of direct and indirect consultations were added as outputs. The range of mean TE scores across the three models was 47-67% in 1998, 50-71% in 1999, 52-72% in 2000, 53-72% in 2001, 52-73% in 2002, 52-75% in 2003, 54-75% in 2004, 58-78% in 2005 and 58-78% in 2006. The mean Malmquist total factor productivity indices for the panel of the 37 CAMHS for *Model PGR *were 1.069 in 1998-2001 and 1.060 in 2001-2004. For Model *PGR_C *these scores were 1.105 in 1998-2001 and 1.151 in 2001-2004.

Amada and Santos [8] assessed the performance of 337 health centres in Portugal in 2005. The inputs were doctors, nurses, and administrative and other staff. The outputs were family planning consultations; maternity consultations; consultations by patients grouped in ages of 0-18, 19-64, and 65 and above; home doctor consultations; home nurse consultations; curatives and other nurse treatments; injections given by a nurse; and vaccinations given by a nurse. The mean TE score was 84.4% (SD = 14.7%).

Marschall and Flessa [9] evaluated the relative efficiency of 20 local health centres in rural Burkina Faso. The inputs chosen were personnel costs, health centre building area (square metres), depreciation of health centre equipment, and vaccination costs in 2004. The health centres' intermediate outputs were number of general consultations and nursing care cases at the dispensary, deliveries in the maternal ward, immunization, and special services such as family planning and prenatal and postnatal consultations. Fourteen health centres were technically efficient and scale efficient. The average TE score was 91% (SD = 17). The mean scale efficiency score was 97% (SD = 12).

Akazili *et al *[10] calculated the TE of 89 health centres in Ghana. The inputs used were non-clinical staff including labourers, clinical staff, beds and cots, and expenditure on drugs and supplies. The outputs were general outpatient plus antenatal care visits, deliveries, children immunized, and family planning visits. Thirty-one (35%) health centres were technically efficient. The inefficient health centres had an average TE score of 57% (SD = 19). Nineteen (21%) health centres were scale efficient, and the inefficient health centres had an average scale efficiency score of 86% (SD = 14).

Milliken *et al *[11] undertook an efficiency comparison of four distinct models of primary health service delivery in Ontario. Their study covered 32 fee-for-service practices (FFS) including family health groups (FHGs), 31 health service organizations (HSOs), 27 family health networks (FHNs) and 19 community health centres (CHCs). The input variables were practice site costs per provider and per patient and provider-patient ratio. The output measures were the average number of visits per patient at the practice site and performance indicators measuring technical quality of care and health service delivery. The study estimated three different scenarios, based on the input measure used: scenario 1 used cost per provider, scenario 2 used cost per patient, and scenario 3 used provider-patient ratio. For scenario 1, the efficiency scores were 60.4% (SD = 16.9) for the entire sample (N = 109), 50.4% (SD = 12.2) for CHCs, 69.1% (SD = 17.2) for FFS, 62.8% (SD = 17.2) for FHNs and 55.9% (SD = 14.4) for HSOs. For scenario 2, the efficiency scores were 43.8% (SD = 22.6) for the entire sample, 25.5% (SD = 16.5) for CHCs, 52% (SD = 22.7) for FFS, 43.9% (SD = 23.3%) for FHNs, and 46.7% (SD = 19.8) for HSOs. Scenario 3's mean technical efficiency score was 41% (SD = 21.1) for the entire sample, 31% (SD = 19.2) for CHCs, 43.9% (SD = 22.4) for FFS, 38.7% (SD = 18.6) for FHNs, and 46% (SD = 21.5) for HSOs.

Kirigia *et al *[12] employed the DEA-based Malmquist productivity index to assess the technical and scale efficiency and productivity change over a four-year period (2001-2004) among 17 public health centres in Seychelles. The inputs used were total number of hours for doctors and for nurses. The outputs were patients dressed, domiciliary cases treated and sum of number of visits for PFMAPIS (pap smear, family planning clinic, maternal and child health, antenatal care and post-natal care, and children immunized and those participating in a school health programme). For the 17 health centres, those that had a variable returns to scale (VRS) TE score of 100% were 10 (59%) in 2001, 9 (47%) in 2002, 9 (53%) in 2003 and 10 (59%) in 2004. The average VRS TE scores were 93%, 92%, 92% and 96%, respectively during the years under consideration. Out of the 17 health centres 5 (29.4%), 6 (35.3%), 7 (41.2%) and 7 (41.2%), respectively, were scale efficient in 2001, 2002, 2003 and 2004. The average scale efficiency score in the sample was 90% in 2001, 93% in 2002, 92% in 2003 and 95% in 2004. The Malmquist index of total factor productivity change (MTFP) was 1.024, technical change was 1.215, efficiency change was 0.843, pure efficiency change was 1.000 and scale efficiency change was 0.843. This meant that health centre productivity increased by 2.4% over the four years, largely due to innovation. Whereas efficiency regressed by 15.7%, technical change (innovation) improved by 21.5% per annum.

Kontodimopoulous, Nanos and Niakas [13] investigated TE of 17 Greek hospital-health centres (HHCs). The inputs used were doctors, nurses and beds, and the outputs were admissions, outpatient visits and preventive medical services. Seven HHCs were technically efficient. The average TE score was 73.23% (SD = 10.09) and the median score was 77.57%.

Masiye *et al *[14] estimated the degree of technical, allocative and cost efficiency among 40 health centres in Lusaka, Central and Copper-Belt provinces of Zambia. Fifty eight per cent were government owned and 42% private-for-profit enterprises. The study used the numbers of clinical officers, nurses and other staff as inputs, and the number of outpatient visits as output. The average TE, allocative efficiency (AE) and cost efficiency (CE) scores for the private health centres were 70%, 84% and 59%, respectively. These scores were 56%, 57% and 33%, respectively, for government health centres. For the whole sample, the averages were 61.9% for TE, 68.5% for AE and 44.5% for CE. Of the 17 private health centres, 5 had a TE score of 100 and 4 had AE and CE scores of 100%. Contrastingly, only 1 of the 23 government health centres had TE, AE or CE scores of 100%.

Renner *et al *[5] investigated TE and SE levels among a sample of 37 public PHUs in Sierra Leone. The six outputs for each PHU were (i) antenatal plus postnatal visits, (ii) child deliveries, (iii) nutritional/child growth monitoring visits, (iv) family planning visits, (v) immunized children under five years and pregnant women immunized with tetanus toxoid (TT), and (vi) total health education sessions conducted through home visits, public meetings, school lectures and outpatient departments. In Sierra Leone PHUs did not provide curative care services but were dedicated to health promotion and disease prevention services. The two inputs were (i) technical staff (community health nurse, vaccinators and maternal and child health aides) and (ii) subordinate staff, including traditional birth attendants, porters and watchmen. Twenty-two (59%) of the 37 health units analysed were found to be technically inefficient with an average score of 63% (SD = 18). On the other hand, 24 (65%) health units were found to be scale inefficient with an average scale efficiency score of 72% (SD = 17).

Osei *et al *[15] estimated the TE of 17 district hospitals and 17 health centres in Ghana in 2000. The DEA model was estimated with four outputs: child deliveries; fully immunized children under the age of five years; maternal visits for antenatal care, postnatal care and family planning, and childcare visits for nutritional and child growth monitoring; and outpatient curative visits. The two inputs were technical staff including medical assistants, nurses and paramedical staff, and support or subordinate staff including cleaners, drivers, gardeners, watchmen and others. Eight (47%) hospitals were technically inefficient with an average TE score of 61% (SD = 12). Ten (59%) hospitals were scale inefficient manifesting an average SE of 81% (SD = 25). Out of the 17 health centres, 3 (18%) were technically inefficient with a mean TE score of 49% (SD = 27) and 8 (47%) were scale inefficient with an average SE score of 84% (SD = 16).

Kirigia, Emrouznejad, Sambo *et al *[16] measured the TE of 32 public health centres in Kenya. The six inputs used were clinical officers and nurses; physiotherapists, occupational therapists, public health officers, dental technologists, laboratory technicians and laboratory technologists; administrative staff; non-wage expenditures; and beds. The four outputs were visits for diarrhoea, malaria, sexually transmitted infections, urinary tract infections, intestinal worms and respiratory disease; visits for antenatal care and family planning; immunizations; and other general outpatient visits. Fourteen (44%) health centres were technically efficient, and the average TE score was 65% (SD = 22). Nineteen (59%) health centres were scale efficient, and the average SE score was 70% (SD = 19).

Linna, Nordblad and Koivu [17] measured the productive efficiency of 228 public dental health centres across Finland. Their study estimated two primal models. Model 1 (*PMODEL1*) was the visit output model whose outputs were total visits to dentists and total visits to hygienists and dental assistants among each of three age groups categorized as 0-18 years, 19-39 years and >39 years. Input variables included number of FTE dentists, number of other employees (hygienists, dental assistants and administrative staff) and total cost of materials and equipment. Model 2 (*PMODEL2*) was the patients' model whose outputs were number of patients treated categorized under three ages groups: 0-18 years, 19-39 years and >39 years. The inputs for Model 2 were as for primal Model 1.

This study, in addition, estimated two cost efficiency models. The cost efficiency Model 1 (*CMODEL1*) used all the outputs used in primal Model 1, plus total operating costs in each health centre as the input variables, while the second model (*CMODEL2*) used all the outputs in primal Model 2, plus total operating costs in each health centre as the input variables. Some 47, 19, 18 and 4 health centres were found to be efficient in *PMODEL1*, *PMODEL2*, *CMODEL1 *and *CMODEL2*, respectively. The average efficiency scores were between 72% and 81% in the primal models, and between 62% and 79% in the cost models.

Kirigia, Sambo and Scheel [18] investigated the TE of 155 primary health care clinics in Kwazulu-Natal Province of South Africa. The clinics were assumed to produce eight types of intermediate outputs, which were visits for antenatal care, child delivery, child health, dental care, family planning, psychiatry services, sexually transmitted diseases and tuberculosis treatment. The inputs included number of nurses and number of general support staff. Forty seven (30%) of the clinics were technically efficient, 25 (16%) of which manifested 100% scale efficiency.

Johnston and Gerard [19] investigated the relative efficiency of 64 (33 large and 31 small) breast cancer screening units in the UK. The outputs were number of invitations, screenings and cancers detected. The inputs were number of FTE radiologists, radiographers, administration staff, medical and nursing staff engaged in assessment work, and number of dedicated mammography machines and assessments performed. The overall sample average TE score was 82.1% (SD = 20) and the median score was 91.2%. Twenty-five units were technically efficient. The average TE score for the large units was 92.1 (SD = 14) and the median was 100%. Thirteen of the large units were efficient. The small units had an average TE score of 84.5% (SD = 84.5) and a median score of 95.6%. Seven of these units were technically efficient.

Salinas-Jimenez and Smith [20] explored the role of quality indicators in primary care and examined the extent to which DEA provides useful insight in the quality of performance of 85 UK family health service authorities (FHSA). The seven indicators of quality used were the number of general medical practitioners per 10 000 patients on a list, the percentage of practices employing a practice nurse, the percentage of general medical practitioners with a patient list of less than 2500, the percentage of general medical practitioners not practising single headedly, the percentage of general medical practitioners who had achieved the higher rate of payments for childhood immunization, the percentage of females aged 35 to 64 registered with the FHSA and who also had an adequate cervical smear in the previous five-and-half years, and the percentage of practice premises that satisfied the minimum standards set out in paragraph 50.10 of the State of Fees and Allowances, excluding practices exempt under paragraph 51.11. The measure of resource inputs for the FHSAs was gross expenditure on general medical services (in British pounds) per head of resident population. Forty-three (51%) of the FHSAs were deemed efficient. Amongst the inefficient units, the average performance level was 92.6%.

The common features of studies reviewed above are: (i) all used DEA approaches to estimate efficiency; and (ii) all used thoroughput measures as proxies of outputs of health facilities. Twelve of the studies estimated TE of primary health care units for one year. Two studies estimated cost efficiencies of PHC units. One study estimated TE, AE and CE. Only two studies attempted to analyse productivity change over a number of years using the DEA-based Malmquist productivity index.

## Methods

### Study area

Sierra Leone is situated on the west coast of Africa, with the North Atlantic Ocean to its west, and lying between Guinea and Liberia. It is divided into 4 major administrative areas (northern, southern, eastern and western regions) and 12 districts (Bo, Mombali, Bonthe, Kailahun, Kambia, Kenema, Koinadugu, Kono, Moyamba, Port Loko, Pujehun, Tonkolili,). Freetown, the capital city, is in the western region. The current study took place in Kailahun and Kenema districts.

The public health system comprises four levels [2]:

#### Peripheral level

PHUs, which are the frontline health services, are classified into three levels:

• At the village level are the MCHPs, which serve less than 5000 people. They are staffed by MCH aides (supported by community health workers, e.g. traditional birth attendants, volunteers) who are trained to provide antenatal care, supervised delivery, postnatal care, family planning, child growth monitoring, immunization, health education, management of minor ailments, and referral of cases to the next level.

• CHPs, which operate in small towns, serve 5000 to 10 000 people and are staffed by state enrolled community health nurses (SECHNs) and MCH aides. They provide similar services as MCHPs in addition to prevention and control of communicable diseases and rehabilitation.

• CHCs, situated at the chiefdom level, cover 10 000 to 20 000 people and are staffed by a community health officer (CHO), SECHNs, MCH aides, an epidemiological disease control assistant and an environmental health officer (EHO). They provide similar services as CHPs in addition to environmental sanitation and supervision of CHPs and MCHPs within their chiefdom.

#### District level

A district hospital is a secondary level facility providing referral support to PHUs. These hospitals provide outpatient, inpatient and diagnostic services, management of accidents and emergencies, and technical support to PHUs. The district health management team (DHMT) is responsible for overall planning, implementation, coordination, monitoring and evaluation of district health services. The DHMT consists of the district medical officer of health, medical officer in charge of the district hospital and officers in charge of various programmes and units.

#### Region level

Regional national hospitals provide tertiary care.

#### National level

The functions of the Ministry of Health and Sanitation include policy formulation, setting standards, quality assurance, resource mobilization, capacity development, technical support, provision of nationally coordinated services such as epidemic control, coordination of health services, and monitoring and evaluation of overall performance and training.

Sierra Leone's population of 5.5 million is served by a total of 1028 health facilities, of which 915 (89%) are owned by the government, 49 (4.8%) by religious bodies, 17 (1.7%) by NGOs and 47 (4.6%) by the private sector. Of the health facilities, 178 (17.3%) are CHCs, 176 (17.1%) are CHPs, 520 (50.6%) are MCHPs, 111 (10.8%) are clinics and 43 (4.2%) are hospitals. All these health facilities are supported by a total of 83 medical specialists (i.e. medical doctors with post-graduate qualification), 118 medical doctors, 1035 nurses, and 1213 other staff (CHOs, cataract surgeons, EHOs, MCH aides, laboratory technicians, pharmacy technicians and vector controllers) [2].

The country spends 4% of about US$1.901 billion annual gross domestic product on health. About 36.4% of the health expenditure comes from the government and 63.6% from private sources. Out-of-pocket payments make up 56.4% of private expenditure on health. Approximately 33.5% of the total expenditure on health comes from external resources for health, i.e. international development partners. In 2006 the per capita total expenditure on health in Sierra Leone was US$ 12, out of which US$ 4 came from the government [21]. It is clear that by 2006 the country had met neither the Abuja [22] target of allocating at least 15% of the national budget to health nor the WHO Commission for Macroeconomics and Health's [23] recommendation of spending at least US$ 34 per capita to provide a package of essential health services. While it is important for the Ministry of Health to keep advocating for allocation of more resources for health development from both domestic and external sources, it is vital to ensure that every dollar allocated is optimally used to provide quality health services for as many people as possible.

The overall health indicators for Sierra Leone are as follows: life expectancy at birth is 49 years, neonatal mortality rate is 45 per 1000 live births, infant mortality rate is 123 per 1000 live births, under-five mortality rate is 194 per 1000 live births, adult mortality rate is 393 per 1000 of the population, maternal mortality ratio is 2100 per 100 000 live births, HIV/AIDS-specific mortality rate is 56 per 100 000 population, malaria-specific mortality rate is 154 per 100 000 of the population, and tuberculosis-specific mortality rate is 140 per 100 000 of the population among HIV-negative people [24].

The 2008 health service coverage was as follows: contraceptive prevalence was 8.2%; antenatal care coverage was 87% for at least 1 visit and 56% for at least 4 visits; 42% of births were attended by skilled health personnel; 28.3% children aged below 5 years were underweight for their age; 97% of neonates were protected at birth against neonatal tetanus; immunization coverage among one-year-olds was 60% for measles, 60% for DTP3, 60% for HepB3 and 64% for Hib3; 25.9% of children aged 6-59 months received vitamin A supplementation; 26% of children aged under 5 years slept under insecticide-treated nets; 30% of children aged below 5 years received an antimalarial treatment for fever; 73.4% of children with diarrhoea received oral rehydration therapy; antiretroviral therapy coverage was 31% for pregnant women and 20% for people with advanced HIV infection; and smear-positive tuberculosis detection rate was 31% using DOTS (directly observed treatment, short-course) with a 89% treatment success rate [24].

The disease risk factors were as follows: population using improved drinking water sources was 49% (86% urban and 26% rural) and using improved sanitation was 13% (24% urban and 6% rural). Some 24% of newborns had low birth weight, 11% of infants were exclusively breastfed for the first six months of life, 37.4% of children were stunted for their age, 21.3% were underweight for their age, and 10.1% of children aged below 5 years were overweight for their age. Alcohol consumption was 6.5 litres of pure alcohol per person per year among adults aged 15 years and older, tobacco use among adolescents aged 13-15 years was 24.1%, and 17% of population aged 15-24 years had comprehensive and correct knowledge of HIV/AIDS [24].

The challenge is whether Sierra Leone can improve the coverage of health services with the current level of health sector investments, especially at the close-to-client PHUs, where the battle to attain national and international health goals (such as the United Nations Millennium Development Goals) will be won or lost. The next section presents the DEA conceptual framework used to explore this issue.

### DEA conceptual framework

**Figure 1:**
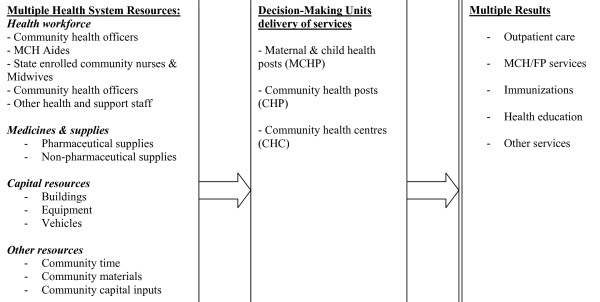
**Relationship between inputs, production process and resulting outputs**.

CHCs, CHPs and MCHPs production processes convert health system *inputs *into health service *outputs*. The relationship among inputs, the production process and resulting outputs is described in Figure 1. Since health centres and health posts employ multiple inputs to produce multiple outputs, we chose to employ the DEA approach, which is versatile in this kind of production scenario.

DEA is a functionalist, linear programming methodology for evaluating relative efficiency of each production unit among a set of fairly homogeneous decision-making units (DMUs) such as MCHPs, CHPs and CHCs. Technical efficiency is a measure of the ability of a DMU to provide maximum quantities of health services (outputs) from a given set of health system resources (inputs). Technical efficiency is affected by the size of operations (scale efficiency) and by managerial practices (non-scale technical efficiency or pure technical efficiency) [25].

DEA plots an efficient frontier using combinations of inputs and outputs from the best performing health facilities. Health facilities that compose the "best practice frontier" are assigned an efficiency score of one (or 100%) and are deemed technically efficient compared with their peers. The efficiency of the health facilities below the efficiency frontier is measured in terms of their distance from the frontier. The inefficient health facilities are assigned a score between zero and one. The higher the score the more efficient a health facility is.

Since MCHPs, CHPs and CHCs employ multiple inputs to produce multiple outputs, their individual TE can be defined as [26]:

The TE score of each MCHP, CHC or CHP in the sample was obtained by solving equations (1) and (2) [16].

Equation 1 is a constant returns to scale input-oriented DEA weights model:(1)

Equation 2 is an input-oriented variable returns to scale DEA weights model:(2)

Where:

∑ - summation

*y_rj _*= the amount of output *r *produced by PHU j,

*x_ij _*= the amount of input *i *used by PHU j,

*u_r _*= the weight given to output *r*, (r = 1,..., t and t is the number of outputs),

*v_i _*= the weight given to input *i*, (i = 1, ..., m and m is the number of inputs),

*n = *the number of PHU, and

j_0 _= the PHU under assessment.

We need to explain what we mean by constant returns to scale and variable returns to scale. Returns to scale refers to the changes in output as all inputs change by the same proportion. For example, if an MCHP, CHC or CHP increased all its health system inputs by the same proportion, the health service outputs might have one of the following outcomes: increase by the same proportion as the inputs, i.e. constant returns to scale (CRS); increase less than proportionally with the increase in inputs, i.e. decreasing returns to scale (DRS); or increase more than proportionally with the increase in inputs, i.e. increasing returns to scale (IRS). Health centres or posts manifesting CRS can be said to be operating at their most productive scale sizes. In order to operate at the most productive scale size, a health facility displaying DRS should scale down both outputs and inputs. If a health facility is exhibiting IRS, it should expand both outputs and inputs in order to become scale efficient [15].

### Output orientation

Managers of MCHs, CHPs and CHCs have no control over inputs, especially staffing. However, given the primary health care orientation of these units, with a strong bias towards health promotion and disease prevention and control, they can influence a great number of people, for example people seeking antenatal and postnatal care, family planning services, birthing services, child growth monitoring, immunization, health education, treatment of common diseases and injuries and vector control (water, sanitation, insecticide treated bed nets) through their public health outreach work among communities. It is for this reason that we estimated an output-oriented DEA model.

### Variables

The DEA models for MCHPs, CHCs and CHPs were estimated with a total of five variables, three of which were outputs and two inputs. The three outputs for each individual health centre were the number of outpatient, maternal, child health and family planning visits, plus immunization visits (OMFE); the number of vector control activities; and the number of health education sessions. The two inputs were the number of community health officers, MCH aides and state enrolled community health nurses (CHO + MA + SECHN); and the number of support staff (including cleaners, drivers, gardeners, watchmen and others). The choice of inputs and outputs for the DEA analysis was guided in part by the availability of data and previous DEA health care studies in the African Region.

### Data

The data used in this study are for 2008. Kailahun and Kenama districts were selected from the 11 districts (i.e. leaving out Pujehun district since a TE study had been conducted there in 2005) using simple random sampling technique. The choice of only two districts (i.e. 18% of the 11 districts where no efficiency study had been carried out) was dictated by research budgetary constraints. Data were collected from all 36 functional MCHPs, 22 functional CHCs and 21 functional CHPs in the two districts using an efficiency questionnaire developed by the WHO African Regional Office for primary health level facilities [27]. Thus, the PHUs surveyed constituted 6.9% of MCHPs, 12.4% of CHCs and 11.9% CHPs in the entire country. The data were analysed using DEAP software developed by Professor Tim Coelli [28].

### Limitations of the Study

Interpretation of the results of this study ought to take cognizance of the limitations of the study. Firstly, the DEA analytical methodology attributes any deviation from the "best practice frontier" to inefficiency, even though some level of deviation could be due to statistical noise such as epidemics, natural disasters, internal displacement of people by civil wars or measurement errors. Secondly, given that DEA is underpinned by a functionalist paradigm using a deterministic/nonparametric technique, it is difficult to use in statistical tests of hypotheses dealing with inefficiency and structure of the production function. Thirdly, it could be argued that the output of MCHPs, CHCs and CHPs is the change in beneficiaries' health status as a result of receiving health services from these institutions. Fourthly, the only health system input used in the current study was health workforce, owing to unavailability of data on non-personnel expenditures. The inputs that were not included in the model include medicines, non-pharmaceutical supplies, buildings, equipment, etc. Fifthly, the study did not give due consideration to social, cultural and behavioural inputs, which can strongly influence the outputs and outcomes of health systems. Lastly, the productivity of the health workforce is likely to be influenced by their emotions, perceptions, cultural background and various human motivation factors that were not captured in this study.

## Results

Table 1 presents the descriptive statistics for the CHCs and CHPs and MCHPs. The average number of outpatient curative and preventive care visits was higher among CHCs than CHPs and MCHPs. This might partially be attributed to the fact that CHCs have higher health workforce endowment than CHPs and MCHPs.

**Table 1:** Descriptive statistics for community health posts, community health centres and maternal, child health and family planning centres

	Community health posts (CHP): n = 21				
	OMFE	Vector control activities	Health education sessions	Number of CHOs + MCH aides + SECHNs	Number of other health staff
Mean	2,604	378	190	1.8	4.2
SD	2,445	402	301	0.4	4.6
Median	1,885.	294	130	2.0	2.0
Min	195	0	2	1.0	0
Max	10,888	1,845	1,482	2.0	15
	**Maternal and child health and family planning (MCHP): n = 36**				
Mean	1,715	319	153	1.2	1.9
SD	947	195	101	0.8	2.9
Median	1,711	346	120	1.0	1.0
Min	60	0	32	0	0
Max	3,566	1,200	452	5	12
	**Community health centres (CHC)**: n = 22				
Mean	4,331	615	230.5	2.5	5.6
SD	2,750	547	325.7	0.9	4.6
Median	3,893	475	128.5	2.0	5.5
Min	22	0	0	1	0
Max	11,268	2,000	1,469	5	15

Note: OMFE is the number of outpatient care visits plus maternal, child health and family planning visits plus immunization visits

### Technical efficiency of MCHPS

Table 2 presents the technical and scale efficiency scores for MCHP clinics. The average score for CRS technical efficiency (CRSTE) was 42.7% (SD = 43.6), for VRS technical efficiency (VRSTE) the average score was 68.2% (SD = 27.2), and for scale efficiency (SE) the average score was 52.8% (SD = 50.6). The average of 68.2% for VRSTE implies that the inefficient MCHPs would need to increase their outputs by 31.8% to become efficient.

**Table 2:** Technical and scale efficiency scores for maternal, child health and family planning clinics

MCHP units	CRSTE	VRSTE	SCALE	Returns to scale
Kpayama	1	1	1	-
Gbo Kakajama	0.782	0.782	1	-
Gbo Lambayama	0.921	0.921	1	-
Woyama	0	0.373	0	DRS
Nyagbebu	0	0.334	0	DRS
Gendema	1	1	1	-
Kondebalihun	0	0.578	0	DRS
Nyandehun Koya	0	0.936	0	DRS
Gbado	0	0.323	0	DRS
Gbagaima	0	1	0	DRS
Sembehun	0	0.784	0	DRS
Gandorhun	0	0.407	0	DRS
Jui	0	0.344	0	DRS
Gelehun	0	0.555	0	DRS
Samai Town	0.619	0.619	1	-
Masahun	0.323	0.323	1	-
Ngelehun	0	0.54	0	DRS
Sandaru Gaura	0.924	0.924	1	-
Konabu	0	0.73	0	DRS
Njagbahun	1	1	1	-
Diamei Dama	0.604	0.604	1	-
Perrie Gaura	0.887	0.887	1	-
Jao Tunkia	0	1	0	DRS
Guala	0.681	0.681	1	-
Semewahun	0.616	0.616	1	-
Sembeima	0.759	0.759	1	-
Ngiehun	1	1	1	-
Bomie	0.838	0.838	1	-
Massayeima	0	0.43	0	DRS
Fola	1	1	1	-
Gbeika	0	0.188	0	DRS
Pendembu Njiegbla	0.44	0.44	1	-
Niahun Gboyama	0	0.557	0	DRS
Ngiehun	1	1	1	-
Jikibu	0.973	0.973	1	-
Mende Buima	0	0.122	0	DRS
**Mean**	**0.427**	**0.682**	**0.528**	
**SD**	**0.436**	**0.272**	**0.506**	
**Median**	**0.382**	**0.706**	**1.000**	

Note: CRSTE = technical efficiency from CRS DEA; VRSTE = technical efficiency from VRS DEA; scale = scale efficiency = crste/vrste

For CRS, of the 36 MCHPs 17 had a TE of 0%, 1 a TE of 31-40%, 1 a TE of 41-50%, 1 a TE of 51-60%, 3 a TE of 61-70%, 2 a TE of 71-80%, 2 a TE of 81-90%, 3 a TE of 91-99%, and 6 a TE of 100%. Thus, as far as CRS was concerned, 30 MCHPs were relatively technically inefficient and the remaining 6 were technically efficient.

For VRS, out of the 36 MCHPs 5 had a TE of 31-40%, 3 a TE of 41-50%, 5 a TE of 51-60%, 3 a TE of 61-70%, 4 a TE of 71-80%, 2 a TE of 81-90%, 4 a TE of 91-99%, and 8 a TE of 100%. Therefore, for VRS, 28 MCHPs were relatively technically inefficient and 8 were technically efficient.

Seventeen MCHPs were scale inefficient and 19 were scale efficient. Nineteen MCHPs manifested constant returns to scale and 17 experienced decreasing returns to scale.

### Technical efficiency of CHCs

Table 3 presents the technical and scale efficiency scores for CHCs. The average score for CRSTE was 62.4% (SD = 32.7), for VRSTE it was 69.2% (SD = 32.7) and for scale efficiency it was 88.8% (SD = 13.5). The mean of 69.2% for VRSTE implies that the inefficient CHCs ought to increase their output by 30.8%.

**Table 3:** Technical and scale efficiency score for community health centres (CHCs)

CHC Units	CRSTE	VRSTE	SCALE	Returns to scale
Dodo	1	1	1	-
Blama	0.14	0.243	0.578	DRS
Baoma Koya	0.214	0.223	0.962	DRS
Levuma	0.767	1	0.767	DRS
Sendumei	1	1	1	-
Boajibu	0.531	0.57	0.932	DRS
Largo	0.296	0.493	0.601	DRS
Bendu	1	1	1	-
Tungai	1	1	1	-
Hangha	1	1	1	-
Ngegbwema	0.879	0.947	0.928	DRS
Tongo	0.876	1	0.876	DRS
Bandajuma	0.576	0.659	0.874	DRS
Pejewa	0.186	0.188	0.992	DRS
Lalehun Kovoma	0.212	0.212	1	-
Dia	0.462	0.509	0.909	DRS
Daru	0.728	1	0.728	DRS
Gbahama	0.306	0.326	0.94	DRS
Kailahun Town	0.82	0.984	0.833	DRS
Pendembu	1	1	1	-
Mobai	0.189	0.283	0.667	DRS
Baiwalla	0.554	0.59	0.939	DRS
**Mean**	**0.624**	**0.692**	**0.888**	
**SD**	**0.327**	**0.332**	**0.135**	
**Median**	**0.652**	**0.803**	**0.936**	

Note: CRSTE = technical efficiency from CRS DEA; VRSTE = technical efficiency from VRS DEA; scale = scale efficiency = CRSTE/VRSTE.

The CRSTE scores for the 22 CHCs were distributed as follows: 3 had a CRSTE of 11-20%, 3 a CRSTE of 21-30%, 1 a CRSTE of 31-40%, 1 a CRSTE of 41-50%, 3 a CRSTE of 51-60%, 2 a CRSTE of 71-80%, 3 a CRSTE of 81-90%, and 6 a CRSTE of 100%. Thus, in the CRS DEA model, 72.3% of the CHCs were found to be technically inefficient.

The distribution of VRSTE scores was as follows: 1 had a VRSTE score of 11-20%, 4 a score of 21-30%, 1 a score of 31-40%, 1 a score of 41-50%, 3 a score of 51-60%, 1 a score of 61-70%, 2 a score of 91-99% and 9 a score 100%. Therefore, in the VRS DEA model 59.1% of the CHCs were technically inefficient.

In terms of SE, the 22 CHCs were distributed as follows: 2 had a SE of 51-60%, 1 a SE of 61-70%, 2 a SE of 71-80%, 3 a SE of 81-90%, 7 a SE of 91-99% and 7 a SE of 100%. Thus, 68.2% of CHCs were scale inefficient.

### Technical efficiency of CHPs

Table 4 portrays the technical and scale efficiency scores for CHPs. The average scores among the CHPs were 57.2% (SD = 35.8) for CRSTE, 59% (SD = 34.7) for VRSTE and 95.5% (SD = 9.4) for scale efficiency. The VRSTE score of 59% indicates that the inefficient CHPs will need to increase their health service output by 41% in order to become technically efficient.

**Table 4:** Technical and scale efficiency score for community health posts (CHP)

CHP Units	CRSTE	VRSTE	SCALE	Returns to scale
Serabu	0.228	0.247	0.923	DRS
Yabaima	0.236	0.37	0.638	DRS
Ngiehun Kojo	1	1	1	-
Konta	1	1	1	-
Jormu	0.405	0.486	0.832	DRS
Benduma	1	1	1	-
Veinema	0.969	0.969	1	-
Kpetema	0.477	0.546	0.874	DRS
Mano Njiegbla	1	1	1	-
Mbowohun	1	1	1	-
Bendu	0.535	0.541	0.989	DRS
Bunumbu	1	1	1	-
Mamboma	0.087	0.087	0.995	-
Mano menima	0.294	0.294	1	-
Bandajuma Kpolihun	0.138	0.138	1	-
Kwellu Ngieya	0.433	0.433	1	-
Ngiehun	0.314	0.314	1	-
Nyandehun	0.404	0.406	0.994	DRS
Mafindor	1	1	1	-
Konjo	0.332	0.414	0.801	DRS
Mano Sewallu	0.15	0.15	1	-
**Mean**	**0.572**	**0.590**	**0.955**	
**SD**	**0.358**	**0.347**	**0.094**	
**Median**	**0.433**	**0.486**	**1.000**	

The 21 CHPs had CRSTE scores distributed as follows: 1 had a CRSTE score of 1-10%, 2 a score of 11-20%, 3 a score of 21-30%, 3 a score of 31-40%, 3 a score of 41-50%, 1 a score of 51-60%, 1 a score of 91-99% and 7 had a score of 100%. Thus, in the CRS DEA model 67% of the CHPs were technically inefficient relative to their peers.

The VRSTE scores among the 21 CHPs were distributed as follows: 1 had a VRSTE score of 1-10%, 2 a score of 11-20%, 2 a score of 21-30%, 2 a score of 31-40%, 4 a score of 41-50%, 2 a score of 51-60%, 1 a score of 91-99% and 7 a score of 100%. Thus, in the VRS DEA model 67% of the community health posts were technically inefficient relative to their peers.

Of the 21 CHPs, 14 had a SE score of 100%. The remaining seven were scale inefficient: 1 had a SE of 61-70%, 1 a SE of 71-80%, 2 a SE of 81-90% and 3 a SE of 91-99%. Seven CHPs manifested DRS, implying that they were too big for their size. The other 14 manifested CRS, indicating that their size was optimal.

## Discussion

### Key findings

The findings show that 28 (77.8%) MCHPs, 14 (59.1%) CHCs and 14 (66.7%) CHPs had VRSTE scores of less 100%, an indication that they were technically inefficient. The average TE scores were 68.2% (SD = 27.2) among MCHPs, 69.2% (SD = 33.2) among CHCs and 59% (SD = 34.7) among CHPs. Thus, the TE of CHCs was higher than that of either MCHPs or CHPs. The average SE scores were 52.8% (SD = 50.6) among MCHPs, 88.8% (SD = 13.5) among CHCs, and 95.5% (SD = 9.4) among CHPs. It is worthy noting that average SE scores for CHPs were higher than those for both CHCs and MCHPs.

The TE of PHUs in Sierra Leone of between 59% and 69.2% was within the ranges for Canada (60.4%) [11], Ethiopia (57%) [6], Ghana (57%, 49%) [10,15], Kenya (65%) [16], Norway (58-78%) [7], Sierra Leone (63%), and Zambia (61.9%) [14]. However, the TE of Sierra Leone's PHUs was lower than those of Burkina Faso (91%) [9], Finland (72-81%) [17], Greece (73.23%) [13], Portugal (84.4%) [8], Seychelles (92-96%) [12] and UK (82.1%, 92.6%) [19,20].

The SE of primary care units in Sierra Leone of between 52.8% and 95.5% was in the same range as those of Ethiopia (95%) [6], Finland (62-79%) [17], Ghana (86%, 84%) [10,15], Kenya (70%) [16] and Sierra Leone (72%) [5]. However, the SE score was lower than that of Burkina Faso of 97% [9].

### Scope of output increases and implications for policy

Were the inefficient MCHPs, CHCs and CHPs to operate as efficiently as their peers on the production possibilities frontier (efficiency frontier), there would be scope to increase health service outputs. Table 5 presents output increases needed to make inefficient MCHPs efficient. The inefficient MCHPs combined would need to increase the number of OMFE visits by 36 848, vector control activities by 7150 and health education sessions by 3660 in order to become efficient.

**Table 5:** Output increases needed to make inefficient maternal and child health posts (MCHP) efficient

MCHP units	OMFE	Vector control activities	Health education sessions
Kpayama	0	0	0
Gbo Kakajama	637	77	51
Gbo Lambayama	221	40	13
Woyama	2,555	399	181
Nyagbebu	2,255	407	199
Gendema	0	0	0
Kondebalihun	1,275	257	215
Nyandehun Koya	2,313	30	23
Gbado	1,081	419	241
Gbagaima	0	0	0
Sembehun	664	126	210
Gandorhun	1,290	583	111
Jui	2,157	381	204
Gelehun	2,116	379	92
Samai Town	929	238	85
Masahun	1,663	460	84
Ngelehun	1,478	241	227
Sandaru Gaura	209	35	13
Konabu	962	231	280
Njagbahun	0	0	0
Diamei Dama	1,008	240	93
Perrie Gaura	338	44	48
Jao Tunkia	0	0	0
Guala	819	168	68
Semewahun	927	231	86
Sembeima	665	118	62
Ngiehun	0	0	0
Bomie	477	55	82
Massayeima	2,223	306	187
Fola	0	0	0
Gbeika	2,195	770	138
Pendembu Njiegbla	1,632	318	137
Niahun Gboyama	1,260	228	182
Ngiehun	0	0	0
Jikibu	74	0	4
Mende Buima	3,425	369	344
**Total**	**36,848 **	**7,150 **	**3,660 **
**Mean**	**1,024 **	**199 **	**102 **
**SD**	**919**	**194**	**95**
**Median**	**928**	**198**	**85**

Table 6 depicts the output increases needed to make inefficient CHCs efficient. To achieve this, the inefficient CHCs combined would need to increase the number of OMFE visits by 70 334 (74%), vector control activities by 4042 (30%) and health education sessions by 7330 (145%).

**Table 6:** Output increases needed to make inefficient community health centres (CHC) efficient

CHC Units	OMFE	Vector control activities	Health education sessions
Dodo	0	0	0
Blama	9,588	344	473
Baoma Koya	7,886	370	470
Levuma	0	0	0
Sendumei	0	0	0
Boajibu	3,812	321	605
Largo	5,946	564	356
Bendu	0	0	0
Tungai	0	0	0
Hangha	0	0	0
Ngegbwema	1,694	51	30
Tongo	0	0	0
Bandajuma	2,448	260	217
Pejewa	8,838	298	630
Lalehun Kovoma	8,614	466	759
Dia	3,991	432	760
Daru	0	0	0
Gbahama	5,631	621	1297
Kailahun Town	97	21	615
Pendembu	0	0	0
Mobai	8,080	0	434
Baiwalla	3,709	294	684
**Total**	**70,334 **	**4,042 **	**7,330 **
**Mean**	**3,197 **	**184 **	**333 **
**SD**	**3,566 **	**215 **	**366 **
**Median**	**2,071 **	**36 **	**287 **

Table 7 portrays the output increases needed to make inefficient CHPs efficient. The inefficient CHPs combined would need to increase the number of OMFE visits by 57 493 (105%), vector control activities by 9688 (122%) and health education sessions by 2966 (74%) in order to become efficient.

**Table 7:** Output increases needed to make inefficient community health posts (CHP) efficient

CHP Units	OMFE	Vector control activities	Health education sessions
Serabu	4,790	1,048	311
Yabaima	2,077	428	162
Ngiehun Kojo	0	0	0
Konta	0	0	0
Jormu	2,146	790	173
Benduma	0	0	0
Veinema	50	12	32
Kpetema	2,395	686	90
Mano Njiegbla	0	0	0
Mbowohun	0	0	0
Bendu	3,097	1,570	75
Bunumbu	0	0	0
Mamboma	6,532	1,671	157
Mano Menima	5,813	705	192
Bandajuma Kpolihun	7,482	938	142
Kwellu Ngieya	5,736	153	124
Ngiehun	4,390	680	442
Nyandehun	4,008	877	263
Mafindor	0	0	0
Konjo	2,177	0	204
Mano Sewallu	6,800	130	599
**Sum**	**57,493**	**9,688**	**2,966**
**Mean**	**2,738**	**461**	**141**
**SD**	**2,659**	**539**	**160**
**Median**	**2,177**	**153**	**124**

In relation to the health units (MCHPs, CHCs and CHPs) with outputs falling short of the variable returns to scale DEA targets, the Ministry of Health and Sanitation could improve their efficiency by boosting demand for underutilized services, i.e. outpatient care, maternal and child health services, family planning services, routine immunization, vector control activities and health education sessions. This might be achieved by leveraging several strategies.

First, the barriers to effective access to health services can be addressed through a number of ways: (i) planned abolishment of official and unofficial user fees in public health facilities [29] which has been shown in Ghana [30-35], Kenya [36,37], Madagascar [38], South Africa [39-42] and Uganda [43-49] to increase health service utilization; (ii) provision of free ambulance services; (iii) improvement of transport in rural areas, where most of primary health care units are situated; (iv) improvement in health workforce motivation and supervision to make them more responsive to non-medical expectations of patients, and by so doing reduce patient waiting, diagnosis and treatment time [50]; (v) implementation of universal coverage policy, which seeks access to key promotive, preventive, curative and rehabilitative health interventions for all residents at an affordable cost, through either tax-funded health services, national social health insurance or a combination of the two [51-54]; (vi) increase of people's access to microfinance and lending programmes to help households to self-insure for consumption of basic services [55]; and (vii) increase demand for underutilized preventive health services by making direct cash transfers to poor households contingent on them utilizing those services [56-58].

Second, the demand for MCHP, CHC and CHP services can be created through leveraging behaviour-change community health programmes to move groups and individuals one step at a time (by providing knowledge, motivation and skills) through the stages (pre-contemplation, contemplation, preparation, action, maintenance) of behaviour change [59].

Third, judicious use should be made of health promotion to stimulate demand for underutilized PHU services. It is important to remember that health promotion is any combination of health education with appropriate legal, fiscal, economic, environmental and organizational interventions aimed at preventing disease [60]. Health promotion action can contribute towards optimal utilization of PHU services by:

• Increasing individual knowledge and skills using health information education and communication (IEC) [61,62];

• Strengthening community action through social mobilization and social marketing [60,62];

• Using mediation and negotiation to create environments that are protective and supportive of health [60,62];

• Developing public health policies, legislation, and fiscal controls that enhance health development [60,62];

• Reorienting health services by emphasizing prevention and promotion of healthy behaviour and lifestyle patterns [62,63].

In short, health promotion methods using IEC, social mobilization, social marketing, mediation, lobbying and advocacy are especially relevant in mobilizing non-health sectors such as agriculture, commerce, culture, education, industry, information technology, sanitation, transport and water to contribute to health development through action on the broad determinants of health [60,62,63].

## Conclusion

This study estimated TE of peripheral health units in Kailahun and Kenema districts of Sierra Leone and the output increases needed to make inefficient units efficient. The findings indicate that 28 (77.8%) MCHPs, 14 (59.1%) CHCs and 14 (66.7%) CHPs were variable returns to scale technically inefficient.

In line with the Ouagadougou Declaration on Primary Health Care and Health Systems in Africa: Achieving Better Health for Africa in the New Millennium [64], there is need to strengthen national and district health management information systems to routinely capture data on health systems input quantities and prices and health services outputs to facilitate regular efficiency analyses. Institutionalization of health facility efficiency monitoring will arm health decision-makers with the vital information needed to take appropriate actions to reduce waste of scarce health systems resources. It will also strengthen health sector advocacy for increasing domestic and external resources for health.

## List of abbreviations

The list of abbreviations include: AE: allocative efficiency; CAMHS: outpatient child and adolescent mental health service units; CE: cost efficiency; CHC: community health centre; CHO: community health officer; CHP: community health post; CRS: constant returns to scale; CRSTE: constant returns to scale technical efficiency; DEA: Data Envelopment Analysis; DHMT: district health management team; DMU: decision-making Units; DOTS: directly observed treatment, short-course; DRS: decreasing returns to scale; FFS/FHGs: family health groups; FHNs: family health networks; FHSA: family health service authorities; FTE: Full-time equivalent; GBP: Great Britain Pound; HSOs: Health service organizations; HEWs: Health extension workers; HHC: Hospital-health centers; IRS: increasing returns to scale; MCHP: maternal and child health posts; MTFP: Malmquist total factor productivity index; NHSSP: national health sector strategic plan; PHUs: peripheral health units; SECHNs: state enrolled community health nurses; SD: standard deviation; SE: scale efficiency; TE: technical efficiency; TT: tetanus toxoid; and VRSTE: variable returns to scale technical efficiency.

## Authors' contributions

JMK, LGS, AR and WA contributed in the design, analysis and writing of all sections of the manuscript. SS and YB contributed in the data collection and writing of some sub-sections of Methods section. All authors read and approved the final manuscript.

## Competing interests

The authors declare that they have no competing interests.

## References

[B1] Government of Sierra Leone, Ministry of Health and Sanitation National health policy 2009 Freetown: Government Printer

[B2] Government of Sierra Leone, Ministry of Health and Sanitation National health sector strategic plan: 2010-2011 2009 Freetown: Government Printer

[B3] Government of Sierra Leone, Ministry of Health and Sanitation Cost-sharing policy 2006 Freetown: Government Printer

[B4] Government of Sierra Leone Hospital Boards Act 2003 Freetown: Government Printer

[B5] Renner Ade Kirigia JM Zere AE Barry SP Kirigia DG Kamara C Muthuri HK Technical efficiency of peripheral health units in Pujehun district of Sierra Leone: a DEA application BMC Health Services Research 2005 5 77 http://www.biomedcentral.com/1472-6963/5/77 10.1186/1472-6963-5-77 PMC1334185 16354299

[B6] Sebastian MS Lemma H Efficiency of the health extension programme in Tigray, Ethiopia: a data envelopment analysis BMC International Health and Human Rights 2010 10 16 http://www.biomedcentral.com/1472-698X/10/16 10.1186/1472-698X-10-16 PMC2893104 20546597

[B7] Halsteinli V Kittelsen SA Magnussen J Productivity growth in outpatient child and adolescent mental health services: the impact of case-mix adjustment Social Science & Medicine 2010 70 439 446 10.1016/j.socscimed.2009.11.002 19926189

[B8] Amado CAEF Santos SP Challenges for performance assessment and improvement in primary health care: the case of the Portuguese health centres Health Policy 2009 91 43 56 10.1016/j.healthpol.2008.11.008 19118917

[B9] Marschall P Flessa S Assessing the efficiency of rural health centres in Burkina Faso: an application of Data Envelopment Analysis Journal of Public Health 2009 17 2 87 95

[B10] Akazili J Adjuik M Jehu-Appiah C Zere E Using data envelopment analysis to measure the extent of technical efficiency of public health centres in Ghana BMC International Health and Human Rights 2008 8 11 http://www.biomedcentral.com/1472-698X/8/11 10.1186/1472-698X-8-11 PMC2605432 19021906

[B11] Milliken O Devlin RA Hogg W Dahrouge S Russell G Comparative efficiency assessment of primary care models using Data Envelopment Analysis Department of Economics Working Paper #0802E 2008 Ottawa (Ontario): University of Ottawa

[B12] Kirigia JM Emrouznejad A Vaz RG Bastiene J Padayachy J A comparative assessment of performance and productivity of health centres in Seychelles International Journal of Productivity and Performance Management 2008 57 1 72 92

[B13] Kontodimopoulous N Nanos P Niakas D Balancing efficiency of health services and equity of access in remote areas in Greece Health Policy 2006 76 49 57 10.1016/j.healthpol.2005.04.006 15927299

[B14] Masiye F Kirigia JM Emrouznejad A Sambo LG Mounkaila A Chimfwembe D Okello D Efficient Management of Health Centres Human Resources in Zambia Journal of Medical Systems 2006 30 473 481 10.1007/s10916-006-9032-1 17233160

[B15] Osei D George M d'Almeida S Kirigia JM Mensah AO Kainyu LH Technical efficiency of public district hospitals and health centres in Ghana: a pilot study Cost Effectiveness and Resource Allocation 2005 3 9 http://www.resource-allocation.com/content/3/1/9 10.1186/1478-7547-3-9 PMC1253524 16188021

[B16] Kirigia JM Emrouznejad A Sambo LG Munguti N Liambila W Using Data Envelopment Analysis to measure the technical efficiency of public health centers in Kenya Journal of Medical Systems 2004 28 2 155 166 10.1023/b:joms.0000023298.31972.c9 15195846

[B17] Linna M Nordblad A Koivu M Technical and cost efficiency of oral health care provision in Finnish health centres Social Science & Medicine 2003 56 343 353 10.1016/s0277-9536(02)00032-1 12473319

[B18] Kirigia JM Sambo LG Scheel H Technical efficiency of public clinics in Kwazulu-Natal province of South Africa East African Medical Journal 2001 78 2 S1 S13 10.4314/eamj.v78i3.9070 12002061

[B19] Johnston K Gerard K Assessing efficiency in the UK breast screening programme: does size of screening unit make a difference? Health Policy 2001 56 21 32 10.1016/s0168-8510(00)00137-8 11230906

[B20] Salinas-Jimenez J Smith P Data envelopment analysis applied to quality in primary health care Annals of Operations Research 1996 67 141 161

[B21] World Health Organization National health accounts: country information. Geneva 2009 http://www.who.int/nha/country

[B22] African Union African Union Summit on HIV/AIDS, Tuberculosis and other related diseases. Decision OAU/SPS/Abuja/3. Addis Ababa 2001

[B23] WHO Macroeconomics and health: investing in health for economic development. Geneva 2001

[B24] WHO World Health Statistics report. Geneva 2010

[B25] Commonwealth of Australia Steering Committee for the Review of Commonwealth/State Service Provision: Data Envelopment Analysis: A technique for measuring the efficiency of government service delivery 1997 Canberra: AGPS

[B26] Charnes A Coopers W Rhodes E Measuring the Efficiency of Decision Making Units European Journal Operational Research 1978 3 429 444

[B27] World Health Organization, Regional Office for Africa Primary Health Care Facility Economics Efficiency Analysis Data Collection Instrument. Brazzaville 2000

[B28] Coelli TJ A Guide to DEAP Version 2.1 A Data Envelopment Analysis (Computer) Program. CEPA Working Paper 8/96 1996 Department of Econometrics, University of New England. Armidale

[B29] Yates R Universal health care and the removal of user fees The Lancet 2009 373 2078 81 10.1016/S0140-6736(09)60258-0 19362359

[B30] Asante FA Chikwama C Daniels A Armar-Klemesu M Evaluating the economic outcomes of the policy of fee exemption for maternal delivery care in Ghana Ghana Medical Journal 2007 41 3 110 117 10.4314/gmj.v41i3.55277 PMC2279082 18470328

[B31] Bosu WK Bell J Armar-Klemesu M Tornui JA Effect of delivery care user fee exemption policy on institutional maternal deaths in the Central and Volta regions of Ghana Ghana Medical Journal 2007 41 3 118 124 10.4314/gmj.v41i3.55278 PMC2279091 18470329

[B32] Penfold S Harrison E Bell J Fitzmaurice A Evaluation of the Delivery Fee Exemption Policy in Ghana: Population estimates of changes in delivery service utilization in two regions Ghana Medical Journal 2007 41 3 100 109 PMC2279083 18470327

[B33] Witter S Adjei S Start-Stop funding, its Causes and Consequences: A Case Study of the Delivery Exemptions policy in Ghana International Journal of Health Planning & Management 2007 22 133 143 10.1002/hpm.867 17623355

[B34] Witter S Aikins M Kusi A Working practices and Incomes of health workers: Evidence from an Evaluation of a Delivery Fee Exemption scheme in Ghana Human Resources for Health 2007 5 2 1 10 10.1186/1478-4491-5-2 PMC1783666 17241454

[B35] Witter S Arhinful DK Kusi A Zakariah Akoto S The experience of Ghana in implementing a user fee exemption policy to provide free delivery care Reproductive Health Matters 2007 15 30 61 70 10.1016/S0968-8080(07)30325-X 17938071

[B36] Mwabu G Mwanzia J Liambila W User charges in government health facilities in Kenya: effect on attendance and revenue Health Policy and Planning 1995 10 2 164 170 10.1093/heapol/10.2.164 10143454

[B37] Mwabu G Wang'ombe J Health service pricing reforms in Kenya International Journal of Social Economics 1997 24 1/2/3 282 293

[B38] Fafchamps M Minten B Public service provision, user fees and political turmoil Journal of African Economies 2007 16 3 485 518

[B39] Bhayat A Cleaton-Jones P Dental clinic attendance in Soweto, South Africa, before and after the introduction of free primary dental health services Community Dentistry and Oral Epidemiology 2003 31 2 105 110 10.1034/j.1600-0528.2003.00006.x 12641590

[B40] Walker L Gilson L We are bitter but we are satisfied: Nurses as street-level bureaucrats in South Africa Social Science & Medicine 2004 59 1251 1261 10.1016/j.socscimed.2003.12.020 15210096

[B41] Wilkinson D Gouws E Sach M Karim SSA Effect of removing user fees on attendance for curative and preventive primary health care services in rural South Africa Bulletin of the World Health Organization 2001 79 7 665 671 PMC2566476 11477970

[B42] Wilkinson D Sach M Karim SSA Examination of attendance patterns before and after introduction of South Africa's policy of free health care for children aged under 6 years and pregnant women British Medical Journal 1997 314 940 941 10.1136/bmj.314.7085.940 PMC2126388 9099118

[B43] Burnham G Pariyo G Galiwango E Wabwire-Mangen F Discontinuation of Cost Sharing in Uganda Bulletin of the World Health Organization 2004 82 3 187 195 PMC2585922 15112007

[B44] Deininger K Mpuga P Economic and welfare impact of the abolition of health user fees: Evidence from Uganda Journal of African Economies 2004 14 1 55 91

[B45] Kajula PW Kintu F Barugahare J Neema S Political analysis of rapid change in Uganda's health financing policy and consequences on service delivery for malaria control International Journal of Health Planning and Management 2004 19 S1 133 153 10.1002/hpm.772 15686066

[B46] Nabyonga J Desmet M Karamagi H Kadama P Omaswa F Walker O Abolition of cost sharing is pro poor: evidence from Uganda Health policy and planning 2005 20 2 101 108 10.1093/heapol/czi012 15746218

[B47] Nabyonga-Orem J Karamagi H Atuyambe L Bagenda F Okuonzi S Walker O Maintaining quality of health services after abolition of user fees: A Uganda case study BMC Health Services Research 2008 8 1 102 10.1186/1472-6963-8-102 PMC2397390 18471297

[B48] Xu K Evans D Kadama P Nabyonga J Ogwang Ogwal P Nabukhonzo P Aguilar AM Understanding the impact of eliminating user fees: Utilization and catastrophic health expenditures in Uganda Social Science & Medicine 2006 62 866 876 10.1016/j.socscimed.2005.07.004 16139936

[B49] Yates J Cooper R Holland J Social protection and health: Experiences in Uganda Development Policy Review 2006 24 3 339 356

[B50] WHO The World Health Report 2006 - working together for health. Geneva 2006

[B51] Carrin G Doetinchem O Kirigia J Mathauer I Musango L Social health insurance: how feasible is its expansion in the African Region? DevIssues 2008 10 2 7 9

[B52] Carrin G Mathaur I Xu K Evans D Universal coverage of health services: tailoring its implementation Bulletin of the World Health Organization 2008 86 11 857 863 10.2471/BLT.07.049387 PMC2649543 19030691

[B53] McIntyre D Garshong B Mtei G Meheus F Thide M Akazili J Ally M Aikins M Mulligan J-A Goudge J Beyond fragmentation and towards universal coverage: insights from Ghana, South Africa and the United Republic of Tanzania Bulletin of the World Health Organization 2008 86 11 871 876 10.2471/BLT.08.053413 PMC2649570 19030693

[B54] WHO The World Health Report: health systems financing - the path to universal coverage. Geneva 2010 10.2471/BLT.10.078741 PMC2878164 20539847

[B55] Leive A Xu K Coping with out-of-pocket health payments: empirical evidence from 15 African countries Bulletin of the World Health Organization 2008 86 11 849 856 10.2471/BLT.07.049403 PMC2649544 19030690

[B56] Morris SS Flore R Olinto P Medina JM Monetary incentives in primary health care and effects on use and coverage of preventive health care interventions in rural Honduras: cluster randomized trial The Lancet 2004 364 2030 37 10.1016/S0140-6736(04)17515-6 15582060

[B57] Lim SS Dandona L Hoisington JA James SL Hogan MC Gakindu E India's Janani Suraksha Yojana, a conditional cas transfer programme to increase births in health facilities: an impact evaluation The Lancet 2010 375 2009 23 10.1016/S0140-6736(10)60744-1 20569841

[B58] Save the Children UK Lasting benefits: the role of cash transfers in tackling child mortality. London 2009

[B59] Jenkins CD Building better health: a handbook of behavioural change 2003 Washington, D.C.: Pan American Health Organization 255 286

[B60] WHO/AFRO Health promotion: a strategy for the African Region. AFR/RC51/12 2003 Brazzaville: WHO/AFRO

[B61] Mushi D Mpembeni R Jahn A Effectiveness of community based safe motherhood promoters in improving the utilization of obstetric care. The case of Mtwara Rural District in Tanzania BMC Pregnancy and Childbirth 2010 10 14 http://www.biomedcentral.com/1471-2393/10/14 10.1186/1471-2393-10-14 PMC2858713 20359341

[B62] WHO Milestones in Health promotion statements from Global Conferences. Geneva 2009

[B63] WHO The Nairobi call to action for closing the implementation gap in health promotion. Geneva 2009

[B64] WHO/AFRO Ouagadougou Declaration on Primary Health Care and Health Systems in Africa: achieving better health for Africa in the new millennium. Brazzaville 2008

